# Interaction of intratumoral microbiota with the tumor immune microenvironment and its impact on cancer progression

**DOI:** 10.3389/fonc.2025.1609889

**Published:** 2025-12-10

**Authors:** Yan Tingting, Zhu Xiaoling, Zhang Yu, Luo Yan, Luo Yang, Shang Xueqin, Tang Shikai

**Affiliations:** 1 Department of Oncology, Affiliated Hospital of Yunnan University, Kunming, China; 2 First Affiliated Hospital of Kunming Medical University, Kunming, China

**Keywords:** tumor microenvironment, intratumoral microbiota, tumor immune microenvironment, immune cells, immunosuppression, antitumor immunity

## Abstract

The intratumoral microbiota, a critical component of the tumor microenvironment (TME), has been demonstrated to significantly impact tumor progression and therapeutic outcomes. Research indicates that intratumoral microbes can affect tumorigenesis, metastasis, and therapeutic response through various mechanisms, such as inducing DNA damage, activating oncogenic signaling pathways, and modulating immune responses. Furthermore, the microbiota exerts dual regulatory effects on the tumor immune microenvironment (TIME), either enhancing anti-tumor immunity or promoting immunosuppression, thereby presenting novel targets for cancer therapy. In this paper, we conduct a review of the origin and composition of the intratumoral microbiota and its dynamic interactions with the TME by synthesizing data from multiple cancer studies. This review elucidates the complex role of the microbiota within the TIME and explores its potential for clinical application.

## 1 Introduction

TME constitutes a dynamic ecosystem comprising tumor cells, immune cells, and stromal components. Traditionally, it has been assumed that the interior of solid tumors is a sterile environment, with the exception of cancers associated with infections, such as human papillomavirus (HPV)-related cervical cancer and *Helicobacter pylori* (*H*. *pylori*)-associated gastric cancer. Since the initial discovery of bacteria within human tumors in the 19th century, advancements in macrogenomic sequencing and spatial imaging technologies have progressively elucidated the presence and functional roles of intratumoral microbiota. Research indicates that distinct microbial communities exist within various solid tumors, including colorectal cancer, breast cancer, lung cancer, and pancreatic cancer. The composition of these communities is closely associated with tumor type, host immune status, and clinical prognosis (1). These microorganisms colonize tumor cells, the extracellular matrix, or immune cells in low biomass forms, with compositions exhibiting organ specificity and tumor type dependency. Notably, while the abundance and diversity of tumor-associated microbes are significantly lower than in normal tissues, their metabolic activity and spatial distribution may influence tumor progression by locally regulating immune responses Nevertheless, the precise mechanisms by which microbiota influence the TIME and their effects on therapeutic response remain subjects of debate. This paper aims to elucidate the biological characteristics of intratumoral microbiota, their mechanisms of action, and their interactions with TIME, thereby providing a theoretical foundation for the development of innovative therapeutic strategies.

## 2 Intratumoral microbial sources and composition

The origins of intratumoral microorganisms remain inadequately characterized, but current evidence indicates that their potential sources primarily include three categories (2–4): (1) sources traversing mucosal barriers, where commensal microorganisms infiltrate the TIME following the disruption of these barriers, as observed in colorectal, pancreatic, and other gastrointestinal tract tumors, as well as lung and cervical cancers; (2) sources originating from adjacent normal tissues; and (3) sources involving hematogenous transmission, wherein microorganisms from the oral cavity, intestines, and other potential sites are transported via the bloodstream to the tumor site, subsequently infiltrating the tumor through compromised blood vessels (5). Furthermore, the hypoxic, nutrient-rich, and immunosuppressive characteristics of the TIME create a conducive environment for the survival of specific microorganisms, such as *E. coli* and *H. pylori* (6).

Given the potential for multiple origins of microorganisms within tumors, it can be postulated that the microbial composition across various cancer types is heterogeneous. This hypothesis was substantiated by a comprehensive study conducted by Ravid Straussman’s team, which analyzed the tumor microbiomes of seven distinct cancer types: lung, breast, pancreatic, ovarian, brain, bone, and melanoma. Their findings revealed that each cancer type possesses a unique microbiome predominantly consisting of intracellular bacteria located within cancer and immune cells (1). Recent studies have also revealed fungal components within tumor microbiomes. The same research team mapped fungal profiles across 35 cancer types, finding that while fungi are present in all cancer types, their abundance and diversity correlate with specific cancer subtypes. Nevertheless, bacteria still dominate the microbial communities within tumors (**Table 1**) (7). This shows that the composition of intratumor microbes is highly heterogeneous and correlates with tumor type, stage and anatomical location. This heterogeneity may influence the biological behavior of the tumor and the response to treatment.

**Table 1:** Microbial diversity within different tumors and their effects on immunity.

Cancer types	Bacterial/Fungal species	Primary metabolites or effector molecules	Effects on the immune system
Pancreatic cancer	*Fusobacterium nucleatum*	Fap2 protein, Adhesin FadA	Fap2 protein binds to TIGIT to suppress immune cell function; FadA activates the β-catenin pathway to promote inflammation and carcinogenesis.
	*Pseudomonas fluorescens*	Cytidine deaminase	Mimicking tumor antigens to activate cross-reactive T cell responses
	*γ-Proteobacteria*	SCFAs	Indirectly leads to immunosuppression (through treatment resistance)
	*Bifidobacterium*	Cell wall polysaccharides	Promoting dendritic cell activation and CD8+ T cell response
	*Malassezia*		Activate the MBL-C3 pathway of the complement system, induce IL-33 secretion, and promote Th2/ILC2 responses.
	*Cladosporium*	Serine protease	Induces IL-33 secretion, promoting Th2-type immune responses
Colorectal cancer	*Clostridium butyricum*	SCFAs	Inhibit IL-6 secretion, reduce M2-type TAM polarization, and enhance CD8+ T cell activity.
	*Fusobacterium nucleatum*	Fap2 protein, Adhesin FadA	Inhibition of NK cell function via the Fap2 protein; activation of the E-cadherin/β-catenin signaling pathway through the FadA adhesion molecule, leading to the recruitment of MDSCs and TAMs.
	*Enterotoxigenic Bacteroides fragilis*		Activate the TLR4/NF-κB inflammatory pathway to induce Th17 inflammatory responses.
	*Streptococcus*		Suppress antitumor immunity, increase MDSCs, TAMs, and neutrophils.
	*Megasphaera elsdenii*	LPS	Induce DC maturation through the LPS/TLR4/IRF4 pathway to promote pro-inflammatory Th1/Th17 cell differentiation.
	*Akkermansia muciniphila*	Tryptophan metabolites	Its absence leads to increased MDSC infiltration; its presence may enhance CD8+ T cell responses.
	*Lactobacillus johnsonii*	Indolepropionic acid (IPA)	Enhancing CD8+ T Cell Memory Function Through H3K27 Acetylation
Esophageal cancer	*Fusobacterium nucleatum*		Activate the TLR4/NF-κB pathway, recruit MDSCs, downregulate CD8+ T cell activity, and form an immunosuppressive microenvironment.
	Porphyromonas gingivalis	gingipains	Induces the release of proinflammatory factors such as IL-6 and IL-8, and upregulates the expression of B7-H4 and KDM5B.
	Streptococcus		Abundance changes correlate with treatment response to immune checkpoint inhibitors.
	*Lactobacillus*		
	*Aspergillus*		Reducing FOXP3+ Treg cell levels may enhance antitumor immunity.
	*Bifidobacteria*		
Oral squamous cell carcinoma	*Fusobacterium nucleatum*		Activation of TLR/CSF-1R induces TAMs to undergo M2 polarization.
	Porphyromonas gingivalis		Directly suppress the function of effector T cells, or promote the proliferation and activity of Tregs; Activation of relevant signaling pathways (such as NF-κB, ERK1/2-Ets1) promotes the EMT process;Promote the release of inflammatory mediators.
Lung cancer	*Bifidobacterium*	Indole-3-acetic acid (IAA)	Activate AHR, downregulate METTL3, and inhibit m6A methylation of STAT3; suppress IL-6, attenuate M2 macrophage polarization, and enhance CD8+ T cell function.
	*Aspergillus sydowii*	β-glucan	Activate IL-1β signaling, recruit MDSCs, and suppress antitumor immunity.
	*Parabacteroides distasonis*		Promote the polarization of TAMs toward the M1 phenotype, enhancing CD8+ T cell activity and infiltration.
	*Cylindrospermopsis Sulfolobus*		Activation of the LCIIAR–miR–ISG15 pathway is associated with an immunosuppressive microenvironment.
	*Akkermansia muciniphila*	SCFAs	Alleviating CD8+ T Cell Exhaustion via the CAF-Neutrophil-CXCL3-PD-L1 Axis.
	*Clostridia, Lachnospiraceae*	SCFAs	Regulating Treg Differentiation via GPR109A and HDACs to Maintain Immune Balance.
	*Gammaproteobacteria, Enterobacteriaceae*	LPS	Promotes chronic inflammation and activates pro-inflammatory signaling pathways such as TLR2/4.
Breast cancer	*Sphingomonas multivorum*	Propionylcarnitine	Recruit Tregs, suppress CD8^+^ T cell function, and promote immune escape.
	*Fusobacterium nucleatum*	Fap2 protein	Inhibiting NK cell activity through the Fap2 protein promotes immunosuppression.
	*Lactobacillus johnsonii*	IPA	Enhancing CD8^+^ T-cell pluripotency to improve the efficacy of immune checkpoint inhibitors
	*Lactobacillus paracasei*	Phytosphingosine	Increase the expression of HLA-I class molecules on tumor cells to enhance CD8^+^ T cell recognition and killing.
Prostate cancer	*Propionibacterium acnes*		Activate the TLR3 signaling pathway, induce IL-6/IL-8 secretion, and drive NF-κB-mediated inflammatory responses.
	*Fusobacterium nucleatum*	Fap2 protein	Inhibition of NK cell activity via the Fap2 protein
	*Shewanella*		Suppressing dendritic cells to increase the number of Treg cells, thereby creating an immunosuppressive environment.
	*Ruminococcus*		Metabolizes androgen precursors and activates the androgen receptor signaling pathway, leading to treatment resistance;Enhancing dendritic cell antigen presentation function to improve CAR-T therapy efficacy.
	*Akkermansia muciniphila*		Associated with response to anti-PD-1 immunotherapy.
	SCFA-producing bacterial communities (such as *Faecalibacterium)*	SCFAs	Dual action: Enhances intestinal epithelial barrier function and reduces inflammation by activating GPR43; induces apoptosis in prostate cancer cells and inhibits EMT by inhibiting HDAC.
Ovarian cancer	*Faecalibaculum rodentium*	Propionate, Butyrate	Activate anti-tumor immunity: Promote the expansion and differentiation of progenitor cell-depleted CD8+ T cells; enhance the efficacy of immune checkpoint inhibitors and PARP inhibitors; facilitate the formation of tertiary lymphoid structures.
	*Acinetobacter seifertii*		Suppression of immune cell function: negatively correlated with M1 macrophages; inhibits macrophage migration capacity, potentially promoting an immunosuppressive microenvironment.
	*Devosia sp* *Ancylobacter pratisalsi*		Risk factor: Associated with poorer disease-specific survival (DSS); its presence often indicates an immunosuppressive microenvironment.
	*Achromobacter deleyi* *Microcella alkaliphila*		Protective factor: Positively correlated with M1-type macrophages; associated with improved patient prognosis, suggesting it may help sustain antitumor immune responses
Cervical cancer	*Lactobacillus*	Lactic acid, H_2_O_2_, bacteriocins, EPS, NO	Enhances mucosal barrier function, promotes macrophage phagocytosis and NK cell cytotoxicity, stimulates antiviral factor secretion, regulates cytokine balance.
	*Prevotella*	Sialidase	Its dominant, highly diverse microbial community may create an immunosuppressive microenvironment.
	*Gardnerella*		May promote chronic inflammatory environment by activating inflammatory pathways such as NF-κB; directly promotes cervical cancer cell proliferation and metastasis; may promote cancer cell metastasis by activating EMT pathways.
Melanoma	*Staphylococcus epidermidis*		Induces tumor-specific CD4+ and CD8+ T cells capable of migrating to distant sites and exhibiting antitumor activity.
	*Bifidobacterium pseudatenulatum* *Roseburia* spp. *Akkermansia muciniphila* *Candida albicans* *Saccharomyces cerevisiae* *Malassezia restricta* *Aspergillus fumigatus* *Chaetomium globosum*		Associated with improved immune therapy response
Gastric cancer	*Fusobacterium nucleatum*		Recruits tumor-associated neutrophils (TANs), promotes their differentiation into pro-tumor subtypes, upregulates PD-L1 expression, leading to CD8_+_ T cell exhaustion and immune escape.
	*Helicobacter pylori*	CagA protein, VacA toxin, small RNA molecules	Induces gastric epithelial cells to express PD-L1; recruits Tregs; inhibits autophagy; downregulates B cell MHC class II molecular pathways; activates Notch signaling in macrophages, collectively forming an immunosuppressive microenvironment.
	*streptococcus anginosus*	Arginine deaminase (ADI)	Metabolizes arginine to ornithine, inhibiting CD8_+_ T cell differentiation and infiltration, promoting tumor growth.
	*Fungi (e.g., Candida, Malassezia)*		May activate inflammasome pathways, recruit immune-suppressive cells (e.g., MDSCs), and inhibit effector T cell function
Renal cell carcinoma	*Aspergillus tanneri*		Inhibits lipid catabolism, upregulates IDO1, VEGFA; Increases exhaustive CD8_+_ T cell infiltration, promotes immunosuppression.
	*Akkermansia muciniphila*	SCFAs	Positively correlated with PD-1 inhibitor response, potentially enhancing immune response
	*Burkholderia cepacia* *Corynebacterium kroppenstedtii*		Synergizes with anti-PD-1 therapy, inhibits tumor growth, enhances antitumor immunity
Liver cancer	*Bacillus subtilis*		Inhibits NK cell ferroptosis, enhances NK cytotoxicity, promotes “cold tumor” to “hot tumor” transformation.
	*Bacteroides ovale*		Inhibits NK cell cytotoxicity (via p-CREB1 mechanism).
	*Butyrate-producing bacteria*	Butyrate	Enhances NK cell migration and function, increases CD107a and IFN-γ secretion.
	*Escherichia*	LPS	Activates TLR4/NF-κB pathway, promotes inflammatory cytokine secretion.
	*Ruminococcus*	SCFAs	Anti-inflammatory, maintains gut homeostasis, modulates anti-tumor immune response
	*Malassezia*		Reduces immune and stromal scores, negatively correlates with inflammatory gene SELE.
	*Candida*		Activates NLRP6 inflammasome; promotes chronic inflammation.
	*Saccharomyces cerevisiae*	β-D-glucan	Inhibits tumor growth by disrupting autophagolysosomal function

## 3 Dual role in cancer development and progression

**Figure 1:**
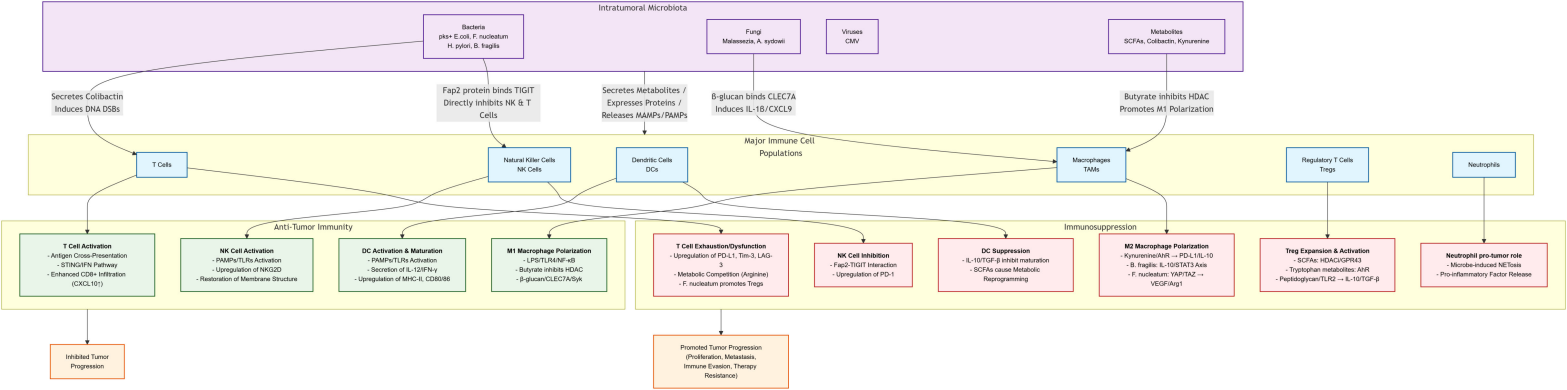
Dual immunomodulatory effects of intratumoral microbiota. The tumor microbiome comprises bacteria, fungi, and other microorganisms that profoundly influence tumor progression and treatment response through complex interactions with tumor cells and immune cells. Its dual nature manifests in two ways: on one hand, certain microorganisms promote immune suppression and accelerate tumor progression; on the other hand, harnessing or modifying specific microorganisms can effectively activate anti-tumor immunity.

Studies have shown that the intratumoral microbiota can influence tumorigenesis, progression and metastasis through multiple mechanisms, while possessing the dual properties of promoting and inhibiting cancer progression (**Figure 1**) (2, 5, 6, 8, 9).

### 3.1 Promoting tumor progression

Intratumoral microorganisms can significantly impact tumorigenesis and progression through various mechanisms. These mechanisms potentially include: (1) induction of genomic instability. Intratumoral microbiota can directly drive tumorigenesis by inducing DNA mutations, disrupting genomic stability, activating oncogenic signaling pathways (such as Wnt/β-catenin and NF-κB), and inducing epigenetic modifications (5, 6, 8, 10, 11). For instance, pks+*E. coli* secretes the genotoxin colibactin, causing DNA double-strand breaks and characteristic mutations (such as APOBEC3B-associated mutations), thereby promoting carcinogenesis (3, 6). *H. pylori* activates carcinogenic pathways (such as Wnt/β-catenin) through abnormal DNA methylation, driving the progression of gastric adenocarcinoma (6). (2) Immune microenvironment remodeling. Microbiota are key regulators of the TME. By activating pathways such as TLR/NF-κB, they promote the release of proinflammatory factors (e.g., IL-6, TNF-α, IL-1β), thereby inducing γδ T cells to secrete IL-17 and IL-22, ultimately establishing a chronic inflammatory environment (2, 6, 10). Additionally, Clostridium difficile can inhibit anti-tumor immunity by activating PD-L1 or recruiting regulatory T cells (Tregs) (3, 6). In pancreatic cancer, the fungal microbiota can promote tumor growth by recruiting TH2 and ILC2 cells and secreting cytokines such as IL-4, IL-5, and IL-13 (12). (3) Metabolic regulation and metastasis. Microbial metabolites can significantly influence tumor progression (13–15). For example, butyric acid influences gene expression and cancer cell differentiation by inhibiting histone deacetylases (HDACs) (3) Microorganisms carried by circulating tumor cells (such as E. coli) can disrupt the vascular barrier and promote the formation of distant metastatic lesions (16). Additionally, Animal model studies confirm that eliminating bacteria within tumors significantly reduces tumor metastasis, suggesting their critical role in the metastatic process (17). These may be related to microbial metabolites.

### 3.2 Potential for tumor suppression

Although related reports are scarce, certain tumor-associated microorganisms have also been found to possess antitumor potential. First, direct killing or suppression of tumors (18). For instance, Neutrophils can limit the growth of tumor-associated microorganisms, indirectly inhibiting tumor progression (19). Engineered tumor-targeting bacteria can be used to deliver therapeutic molecules such as interferon-β, reshaping the TIME and suppressing tumor growth and metastasis (20). Second, activating antitumor immunity: Certain bacteria can induce IFN-γ production by activating the STING signaling pathway or releasing outer membrane vesicles, thereby activating T cells and NK cells to enhance antitumor immunity (5, 21). Additionally, certain tumor-associated microorganisms exert indirect effects analogous to “gut probiotics,” potentially exerting antibacterial effects through mechanisms such as antigen presentation and immune response polarization. However, their precise mechanisms within tumors require further investigation (5), potentially involving the activation or inhibition of signaling pathways including ROS, β-catenin, TLR, ERK, NF-κB, and STING (22–24).

## 4 Relationship between intratumoral microorganisms and the tumor immune microenvironment

Intratumor microorganisms not only play a role in tumorigenesis and development, but may also influence the TIME. Studies have shown that these microorganisms interact with immune cells through multiple mechanisms and exert bidirectional regulatory effects on the TIME, which may either enhance the anti-tumor immune response or promote immunosuppression, thus affecting the therapeutic efficacy (**Figure 1**).

### 4.1 Role of intratumoral microorganisms on immune cells

#### 4.1.1 T cells

Intratumoral microorganisms can suppress T cell function by inducing T cell exhaustion, metabolic competition, and inflammation in the microenvironment. Research indicates that certain tumor-associated microbes create an immunosuppressive microenvironment by secreting metabolic byproducts or inducing immunosuppressive cytokines (such as IL-10 and TGF-β), thereby inhibiting T cell activation and proliferation (1, 25). Concurrently, they enhance the suppressive capacity of Tregs by depleting key metabolites like arginine, ultimately suppressing antitumor immune responses (26). This metabolic competition not only impairs effector T cell function but may also lead to premature T cell exhaustion (27). Furthermore, tumor-associated bacteria can promote immune evasion by upregulating PD-L1 expression or inducing T cell exhaustion phenotypes (e.g., Tim-3, LAG-3) (28).

Intratumoral microorganisms have been shown to activate T cell-mediated antitumor immune responses through several mechanisms. First, microbial antigens can induce cross-reactive T cell activation through a “molecular mimicry” mechanism. For instance, functional intratumoral bacteria (such as the AUN strain isolated in this study) can promote tumor-specific T cell killing by releasing pathogen-associated molecular patterns (PAMPs), activating dendritic cells (DCs), and enhancing antigen presentation (28). *Escherichia coli* and *Bifidobacterium bifidum* can secrete antigens or metabolites (such as SCFAs) to activate CD8_+_ T cells via dendritic cells, thereby enhancing their cytotoxic function (5, 29). Secondly, intratumoral microorganisms can potentiate the efficacy of immune checkpoint inhibitors by activating the interferon signaling pathway through pattern recognition receptors such as Toll-like receptors (TLRs) and STING, which in turn upregulates PD-L1 expression (5). Finally, intratumoral microorganisms can enhance T-cell infiltration. For example, *Fusobacterium nucleatum* promotes CD8_+_ T-cell recruitment in colorectal cancer by modulating chemokines (e.g., CXCL10) and inhibits PD-1 expression on CD8+ TILs and reactivates their effector function (30, 31).

#### 4.1.2 Regulatory T cells

Intratumoral microorganisms may influence the number or function of Tregs by releasing metabolic products. Initially, intratumoral microbes mediate Treg expansion through metabolic products. For instance, SCFAs promote Treg differentiation and function by inhibiting HDACs or activating G protein-coupled receptors (such as GPR43). They also maintain the immunosuppressive phenotype of Tregs by enhancing the epigenetic stability of the Foxp3 gene (32). *Lactobacillus* metabolizes tryptophan to produce indole-3-propionic acid, which directly induces Treg differentiation and enhances their suppressive activity by activating the AhR (33, 34). Furthermore, Peptidoglycan or LPS released by Gram-positive bacteria activate pattern recognition receptors on Treg surfaces (e.g., TLR2, NOD2), inducing them to secrete IL-10 and TGF-β, thereby enhancing immunosuppressive functions (35, 36). These studies suggest that the microorganisms and their metabolites have complex mechanisms of action in regulating Tregs function, which are important for understanding and improving cancer immunotherapy.

#### 4.1.3 Macrophages

Recent studies have revealed that intratumoral microorganisms significantly affect the functional polarization and anti-tumor immune responses of TAMs mainly through metabolic regulation, immune modulation and signaling pathway activation.

Firstly, reshaping the metabolic characteristics of TAMs through microbial metabolites primarily involves SCFAs regulating epigenetic reprogramming. For instance, butyrate suppresses HDACs, upregulates M1-type markers (such as iNOS and IL-12), and promotes TAM polarization toward the anti-tumor M1 phenotype (37, 38). Propionic acid activates G protein-coupled receptor 43 (GPR43), inhibits STAT6 phosphorylation, and blocks IL-4/IL-13-induced M2 polarization (39, 40). Furthermore, other metabolites exert similar effects. For example, E. coli metabolizes tryptophan into kynurenine, which activates the aryl hydrocarbon receptor (AhR). This induces TAMs to express PD-L1 and IL-10, promoting M2 polarization while suppressing CD8+T cell function (41, 42). Secondary bile acids (e.g., deoxycholic acid) inhibit the NF-κB pathway via the farnesoid X receptor (FXR) and the membrane receptor TGR5, reducing proinflammatory cytokine (TNF-α, IL-6) secretion by TAMs and enhancing M2 function (43).

Secondly, microorganisms directly activate pattern recognition receptors (PRRs) to promote the exertion of immune regulatory effects. For instance, lipopolysaccharide (LPS) released by *Fusobacterium nucleatum* activates the MyD88/NF-κB pathway in TAMs via TLR4, promoting M1 polarization (44, 45). β-glucan from *Malassezia* binds CLEC7A (Dectin-1), activating the Syk/CARD9 pathway to induce TAMs to secrete IL-1β and CXCL9, recruiting Th1 cells and enhancing antitumor immunity (46). Furthermore, bacterial DNA or mitochondrial DNA activates the cGAS-STING pathway in TAMs, promoting type I interferon (IFN-β) secretion, enhancing M1 polarization, and boosting antigen presentation capacity (47).

Additionally, TAMs polarization is regulated by activating signaling pathways—core hubs in microbe-host interactions—including STAT3/STAT1 balance regulation, the Wnt/β-catenin pathway, and the Hippo-YAP pathway. Research indicates that *Bacteroides fragilis* activates the IL-10/STAT3 axis by secreting polysaccharides, thereby inhibiting STAT1 phosphorylation, suppressing M1 polarization, and promoting the proangiogenic phenotype of TAMs (2, 48). Butyrate inhibits β-catenin degradation, upregulates Wnt pathway target genes (e.g., CCL2), and promotes the transition of TAMs to immunosuppressive M2-type (49). *Clostridium difficile* activates the YAP/TAZ signaling pathway, inducing TAMs to express VEGF and arginase-1 (Arg1), thereby promoting tumor angiogenesis and immune evasion (50, 51).

#### 4.1.4 Dendritic cells

Intratumoral microorganisms alter the maturation, metabolism, and functionality of DCs via multiple pathways, leading to a bidirectional modulation—either activation or inhibition—of tumor immunity. Empirical evidence indicates that the activation of DCs by intratumoral microorganisms enhances their antigen-presenting capabilities and the secretion of proinflammatory molecules.

On the one hand, microbes may activate pattern recognition receptors (e.g., TLRs, NOD-like receptors) on the surface of DCs by releasing PAMPs (e.g., LPS, flagellin), thereby promoting the maturation and antigen-presenting capacity of DCs (52, 53). For example, the activation of TLR4 leads to the upregulation of MHC-II-like molecules and co-stimulatory molecules, such as CD80 and CD86, on the surface of DCs, thereby enhancing their proficiency in presenting tumor antigens (28, 54). On the other hand, research indicates that microorganisms or their metabolites can stimulate DCs to produce pro-inflammatory cytokines, such as IL-12 and IFN-γ, thereby activating T-helper 1 (Th1) immune responses and enhancing the anti-tumor efficacy of CD8+T cells (55, 56). The synergistic interaction between IL-12 and IFN-γ further contributes to the persistence and potency of anti-tumor immune responses by modulating immune cell dynamics within the tumor microenvironment (57, 58). Moreover, intratumoral microorganisms can impair DC function by inducing functional depletion and altering their metabolic states. Some intratumoral microbes may impede DC differentiation and maturation by secreting inhibitory factors, such as IL-10 and TGF-β, or by promoting VEGF secretion by tumor cells (56, 59, 60). Additionally, microbial metabolites (e.g., SCFAs) may influence the immune activation capacity of DCs by modulating their metabolic pathways, such as glycolysis and oxidative phosphorylation (61, 62).

#### 4.1.5 Natural killer cells

NK cells play a crucial role in tumor immune surveillance, and their function may be directly or indirectly influenced by microorganisms within the TIME (63). On the one hand, intratumoral microorganisms can directly acton NK cells, enhancing their immune recognition capabilities and restoring the structure and function of NK cell membranes. Literature indicates that specific intratumoral microbes, such as functional bacteria A-gyo, UN-gyo, and AUN isolated from solid tumors, can activate pattern-recognition receptors on NK cells through the release of immunogenic signaling molecules, thereby improving the recognition of tumor cells (64). For instance, these functional bacteria facilitate the formation of immune synapses between NK cells and tumor cells by upregulating the expression of activating receptors, such as NKG2D, on NK cells or by inducing the expression of NKG2D ligands on tumor cells. This interaction leads to the release of granzymes and perforin, which directly kill tumor cells and enhance the cytotoxic efficiency of NK cells against tumor cells (28, 65). At the same time, intratumoral microbes have the potential to restore the sphingolipid content of NK cell membranes and reconstruct the membrane protrusion architecture. This is achieved by modulating the local metabolic milieu, such as through the supplementation of sphingomyelin or the inhibition of sphingomyelinase activity, thereby augmenting the NK cells’ ability to recognize and eliminate tumor cells (66, 67).

In addition, microbes may exert an indirect influence on NK cells by reshaping the TIME and mitigating tumor-induced immunosuppression. Empirical evidence suggests that intratumoral microbes can modulate NK cell function indirectly through the secretion of metabolites or signaling molecules, such as IL-10, which in turn affect the activity of TAMs, Tregs, and DCs within the microenvironment (68–72). For instance, research has demonstrated that synthetic bacteria can enhance the anti-tumor immune response of NK cells by inhibiting neutrophil activity via IL-10 signaling while simultaneously activating CD8+ T cells (66). Additionally, microbes can augment NK cell activity by promoting the secretion of pro-inflammatory cytokines, such as IL-12 and IFN-γ (28). Within the tumor microenvironment, NK cells frequently experience functional suppression due to the upregulation of inhibitory receptors, such as PD-1 and cytotoxic T-lymphocyte-associated protein 4 (CTLA-4), or the presence of inhibitory cytokines, including TGF-β and IL-10 (73). Intratumoral microbes may block immunosuppressive signals and restore NK cell killing function through competitive depletion of inhibitory factors or secretion of immunostimulatory molecules (e.g., CpG sequences in bacterial DNA) (28, 66).

#### 4.1.6 Neutrophils

The interaction between intratumoral microbes and neutrophils constitutes a complex and multifaceted process that encompasses various immune responses and cell signaling pathways. Within the cancer microenvironment, neutrophils transcend their traditional role as mere immune cells; they actively contribute to tumor progression and metastasis. The behavior of neutrophils in this context is significantly influenced by the presence of microorganisms. Microbes possess the ability to modulate neutrophils function through direct interaction. For instance, some bacteria can evade immune clearance by delaying the fusion with neutrophils granules, thereby enhancing their survival within these cells (74). Furthermore, the presence of microorganisms can impede T cell infiltration by inducing neutrophils to release extracellular traps (NETs), which form a physical barrier, while simultaneously releasing pro-inflammatory factors that exacerbate inflammation within the TME (75).

### 4.2 Promotion of anti-tumor immunity by intratumoral microorganisms

Research has demonstrated that intratumoral microorganisms can augment anti-tumor immune responses through various mechanisms, including the modulation of immune system pathways and cancer or immune-related signaling pathways. Within the TME, microorganisms can enhance anti-tumor immunity by activating the cGAS-STING signaling pathway. These intratumoral microbes can stimulate the STING signaling pathway, thereby enhancing the activity of T cells and NK cells, promoting the formation of tertiary lymphoid structures (TLS), and facilitating antigen presentation (76). *Bacteroides fragilis* produces STING agonists that induce anti-tumor macrophage polarization, promote natural killer cell activation, and enhance interactions with DCs (22). *Lactobacillus strains* induce cGAS/STING-dependent type I interferon production, activate dendritic cells, and recruit tumor-specific CD8 T cells to the TME (23, 77). Collectively, these mechanisms contribute to the enhancement of the body’s anti-tumor immune response, thereby inhibiting tumor growth and metastasis.

Intratumoral microbes may also modulate immune responses by influencing the TME. Research indicates that these microbes can alter the TME by modulating the infiltration and activity of immune cells, thereby impacting the immune characteristics of the tumor (78). For example, specific microorganisms (*Saccharomyces cerevisiae*, *Pseudomonas pseudomallei*, and *Streptomyces* spp.) present in pancreatic adenocarcinoma (PDAC) tissues enhance the activity of CD8 T cells and granzyme B cells, thereby triggering potent antitumor immune responses and improving overall survival rates (79). The intratumoral microbiome (including *Clostridiales* strains, EBV, and HBV) induces chemokine production, enhancing CD8 T cell infiltration into tumor tissues and thereby improving survival rates in melanoma patients (80). And microbes may enhance immune surveillance through the secretion of cytokines or chemokines, such as CXCL9/10, which recruit effector immune cells, including CD8+ T cells and dendritic cells, to the tumor site (81). The presence of specific bacterial communities is strongly correlated with the quantity and activity of tumor-infiltrating lymphocytes, suggesting that microorganisms may play a crucial role in regulating the TIME (82).

The presence of intratumoral microbes has been associated with the tumor’s response to therapeutic interventions. Empirical evidence indicates that these microbes can affect the efficacy of immunotherapy by modulating the host’s immune system. Specifically, Microorganisms can modulate the tumor microbiome by directly migrating into tumors and altering the tumor microenvironment, thereby enhancing the efficacy of immune checkpoint inhibitor (ICI) therapy (83). Furthermore, intratumoral microbes may augment the impact of immunotherapy by influencing the function and activity of immune cells (84). In summary, the roles of intratumoral microorganisms in antitumor immunity are complex and multifaceted. They may potentiate antitumor immune responses not only through the direct activation of immune cells but also indirectly by modulating the TME and affecting the outcomes of immunotherapy. These insights provide a crucial theoretical foundation and direction for future research aimed at developing novel cancer immunotherapy strategies (85).

### 4.3 Intratumoral microorganisms as drivers of immunosuppression

The bacterial communities within tumors exert a dual effect on cancer progression. On one hand, they enhance antitumor immunity, thereby aiding in the resistance against the growth and spread of cancer cells. On the other hand, they promote cancer progression through immunosuppressive mechanisms, creating favorable conditions for the survival and proliferation of cancer cells (86).

Intratumoral microorganisms can shape immunosuppressive microenvironments through complex interactions with the host immune system, thereby influencing the tumor’s capacity for immune evasion (87). Microbial communities within tumor tissues can directly suppress immune cell activity through the production of immunomodulatory molecules, or indirectly facilitate immunosuppression by modifying signaling pathways within host cells (88). For example, bacteria in colorectal cancer secrete IL-17, promoting cancer cell growth by increasing B cell infiltration in tumor tissue (19). Symbiotic bacteria stimulate γδT cells to produce IL-17, enhancing local inflammatory responses in lung cancer and driving tumor progression (89). In PDAC, the microbiota modulates TAMs via TLR signaling pathways, creating an immunosuppressive TME (90).

In addition, intratumoral microorganisms have been shown to facilitate the expansion of immunosuppressive cell populations, contributing to the formation of immunosuppressive TME. Research indicates that these microorganisms can promote the proliferation of cells such as MDSCs, Tregs, and M2-type TAMs (90). For instance, *Aspergillus sydowii* has been observed to enhance the expansion and activation of MDSCs and TAMs via the IL-1β-mediated β-glucan/Dectin-1/CARD9 signaling pathway. This process results in the inhibition of cytotoxic T-lymphocyte (CTL) function and the aggregation of PD-1+ CD8+ T-cells, thereby contributing to the progression of lung adenocarcinoma (91). Additionally, Fusobacterium nucleatum impairs the anti-tumor activity of NK cells and T cells by interacting with TIGIT through the Fap2 protein (3). Some intratumoral microorganisms (*B. fragilis* and *Fusobacterium*) also increase ROS levels or produce metabolites such as short-chain fatty acids, which promote the proliferation of Tregs and MDSCs. These cells, in turn, secrete inhibitory factors like IL-10 and TGF-β, thereby diminishing the antitumor efficacy of effector T cells (92, 93).

In various studies, a decrease in microbial diversity or the enrichment of specific microbial populations is frequently linked to the development of an immunosuppressive TIME. For instance, in esophageal squamous cell carcinoma (ESCC), a high Shannon index, indicative of microbial diversity, has been correlated with diminished PD-L1 upregulation and reduced infiltration of NK cells and activated CTLs. This observation suggests that microbial diversity may facilitate immunosuppression (94). Similarly, in PDAC, intratumoral microbes have been shown to inhibit the differentiation of CD4+ T cells and Th1 cells through the activation of TLRs and to impede the polarization of M1 macrophages (90).

In summary, intratumor microbes play a dual role in regulating the tumor immune microenvironment, both by promoting anti-tumor immune responses and possibly by inducing immunosuppression to promote tumor progression. Future studies should further explore how the properties of intratumor microbes can be exploited to improve the efficacy of tumor immunotherapy.

## 5 Clinical translation and application potential

The dual role of tumor-associated microbiota and its aforementioned effects on immune cells determine its immense clinical application potential. Several studies have reported the potential of tumor-associated microbiota in translational clinical applications.

### 5.1 Association with clinical prognosis

The first is that changes in intratumoral microbial diversity correlate with patient prognosis. Studies have shown that microbial alpha diversity in ESCC tumor tissues is significantly lower than in normal tissues, and patients with high bacterial abundance have a worse prognosis (94). The β-diversity characteristics of the intratumoral microbiome exhibit significant differences and can predict the therapeutic efficacy of neoadjuvant chemoimmunotherapy (NACI) efficacy, for example, *Streptococcus* bacteria accumulate in esophageal squamous cell carcinoma (ESCC), tumor-infiltrating CD8+ T cells increase, and respond well to anti-PD-1 therapy (2). However, the impact of increased or decreased microbial α and β diversity on clinical prognosis varies across different tumor types, and even the same microbial species may exhibit differing effects across distinct tumors.

Secondly, certain specific microbial taxa possess distinct prognostic significance in related tumors. For instance, the presence of *Lactobacillus* is an independent predictor of diminished survival in ESCC patients (94). Similarly, *Fusobacterium nucleatum* is linked to adverse prognosis and chemotherapy resistance in esophageal cancer (95). Conversely, an enrichment of Streptococcus is correlated with extended disease-free survival and an improved response to anti-PD-1 therapy in ESCC patients (2).

### 5.2 Potential applications in tumor therapy

The potential application of intratumoral microorganisms in tumor therapy has attracted much attention in recent years. Research indicates that these microorganisms can impact tumorigenesis, progression, and therapeutic response through various mechanisms. Specifically, intratumoral microbes have the capacity to modulate the immune microenvironment within tumors and affect anti-tumor immune responses, thereby presenting potential novel targets for therapeutic intervention (76).

Current research is concentrated on two primary areas: predicting treatment response and developing intervention strategies targeting the microbiota. For instance, the characterization of intratumoral streptococci has been shown to predict response to NACI in patients with ESCC (2). In locally advanced rectal cancer (LARC), variations in microbial alpha and beta diversity, along with 12 distinct microbial taxa (such as *Faecalitalea* and *Collinsella*), have been linked to immune cell infiltration in patients achieving pathologic complete remission (pCR) compared to those not achieving pCR (96). Intervention strategies targeting microbes include colony transplantation, dietary and metabolic modifications, and microbial ablation. The literature indicates that mice receiving fecal microbial transplantation from responders or streptococcal colonization exhibit increased intratumoral CD8+T cell infiltration and enhanced efficacy of anti-PD-1 therapy (2). Additionally, a high-fiber diet has been found to improve immune checkpoint blockade (ICB) efficacy by activating the intratumoral IFN-I-NK-DC axis (22). The elimination of intratumoral microorganisms in PDAC has been shown to remodel the TME, reverse immunosuppression, and enhance the response to immunotherapy (90).

Microbial therapies are undergoing significant advancements, with researchers investigating the potential of employing microorganisms as vehicles for drug delivery and as therapeutic agents. For instance, bacteria can be genetically engineered to express proteins with therapeutic properties, facilitating the targeted delivery of anti-cancer proteins directly within tumor sites (97). Furthermore, bacteria have the capability to impede tumor progression by disrupting the vasculature within tumor tissues and inducing thrombosis, thereby obstructing the nutrient supply to tumor cells (98).

In conclusion, the potential application of intratumoral microbes in tumor therapy provides new perspectives and strategies for cancer treatment. With in-depth studies on the mechanisms of interaction between intratumoral microbes and tumors, more effective cancer therapies are expected to be developed in the future.

## 6 Summary

Although the role of microbiota in the regulation of TIME has been widely validated, contradictions remain between different studies. For example, microbiota in PDAC can either drive immunosuppression (90) or activate anti-tumor immunity through specific genera (79). Such differences may be related to microbial composition, host genetic background and tumor heterogeneity. Future combination of multi-omics technologies (e.g., single-cell sequencing, spatial transcriptome) is needed to deeply analyze the microbe-TIME interaction network and develop microbiota-based precision therapeutic strategies.

.
